# A proteomic approach to the development of DIVA ELISA distinguishing pigs infected with *Salmonella* Typhimurium and pigs vaccinated with a *Salmonella* Typhimurium-based inactivated vaccine

**DOI:** 10.1186/s12917-016-0879-1

**Published:** 2016-11-11

**Authors:** Jan Gebauer, Hana Kudlackova, Marcel Kosina, Kamil Kovarcik, Radek Tesarik, Alena Osvaldova, Martin Faldyna, Jan Matiasovic

**Affiliations:** 1 Department of Immunology, Veterinary Research Institute, Hudcova296/70, 62100 Brno, Czech Republic; 2 Department of Experimental Biology, Faculty of Science, Masaryk University, Kotlarska267/2, 611 37 Brno, Czech Republic; 3 Bioveta a. s., Komenskeho212/12, 683 23 Ivanovice na Hane, Czech Republic; 4 Department of Virology, Veterinary Research Institute, Hudcova296/70, 62100 Brno, Czech Republic; 5 Faculty of Veterinary Medicine, University of Veterinary and Pharmaceutical Sciences, Palackeho 1/3, 612 42 Brno, Czech Republic

**Keywords:** Porcine, *Salmonella* Typhimurium, DIVA vaccine, Recombinant protein, Mass spectrometry

## Abstract

**Background:**

*Salmonella enterica* serovar Typhimurium is one of the most common enteropathogenic bacteria found in pigs in Europe. In our previous work, we demonstrated the protective effects in suckling piglets when their dams had been vaccinated with an *S*. Typhimurium-based inactivated vaccine. This study is focused on a procedure leading to serological discrimination between vaccinated and infected pigs. As we supposed, distinct environment during natural infection and in bacterial cultures used for vaccine preparation led to a slightly different spectrum of expressed *S*. Typhimurium proteins. The examination of porcine antibodies produced after the experimental infection with *S*. Typhimurium or after vaccination with *S*. Typhimurium-based inactivated vaccine by affinity chromatography and mass spectrometry revealed differences in antibody response applicable for serological differentiation of infected from vaccinated animals.

**Results:**

Antibodies against *Salmonella* SipB, SipD and SseB proteins were detected at much higher levels in post-infection sera in comparison with control and post-vaccination sera. On the other hand, proteins BamB, OppA and a fragment of FliC interacted with antibodies from post-vaccination sera with a much higher intensity than from control and post-infection sera. In addition, we constructed ELISA assays using post-infection antigen - SipB protein and post-vaccination antigen - FliC-fragment and evaluated them on a panel of individual porcine sera.

**Conclusions:**

The analysis of antibody response of infected and vaccinated pigs by proteomic tools enabled to identify *S*. Typhimurium antigens useful for distinguishing infected from vaccinated animals. This approach can be utilized in other challenges where DIVA vaccine and a subsequent serological assay are required, especially when genetic modification of a vaccine strain is not desirable.

**Electronic supplementary material:**

The online version of this article (doi:10.1186/s12917-016-0879-1) contains supplementary material, which is available to authorized users.

## Background

Infections caused by non-typhoid *Salmonella enterica* serovar Typhimurium (*Salmonella* Typhimurium) constitute a persistent problem in human and veterinary medicine. *Salmonella* Typhimurium is the most frequent serotype found in pigs. Contaminated pork and porcine products are thus a source of infection for human consumers [1]. A possible way to moderate the *Salmonella* burden in pigs is vaccination. A successful and widely used vaccine should allow distinguishing vaccinated animals from those that were naturally infected, so-called DIVA approach (Differentiating Infected from Vaccinated individuals) [2]. Available diagnostic serological tests for the evaluation of *Salmonella* infections in pigs are based on measurements of the level of antibodies induced by O-antigens, the outer segment of bacterial lipopolysaccharide (LPS) [3]. These assays do not allow us to distinguish infected and vaccinated animals when *Salmonella* strain without any deletion in genes responsible for lipopolysaccharide formation is used for vaccination [4]. On the other hand, LPS plays a role as an inducer of the immune response, which might be beneficial for the vaccination itself [5, 6]. Selke et al. [7] introduced a live negative-marker vaccine based on *Salmonella* Typhimurium strain with deleted gene for the outer membrane protein *ompD*. Bearson et al. [8] prepared a similar attenuated live negative-marker vaccine with a deletion of multiple small regulatory RNAs combined with *rfaH* mutation. However, using a genetically modified live bacterial strain as a vaccine may be questionable because of current regulation and public non-acceptance of genetically modified organisms in Europe.

With an unmodified inactivated *Salmonella* Typhimurium-based vaccine for pigs developed in our previous work we achieved a similar level of protectivity for suckling piglets [9] as Selke et al. [7]. In this study, we extend the development of this vaccine to include DIVA testing. We took advantage of the fact that *Salmonella* express virulence factors (proteins from SPIs - *Salmonella* pathogenicity islands) in an environment-dependent manner. We thus expected differences in bacterial protein expression under in vitro and in vivo conditions. We analysed the antibody response to the vaccine based on inactivated *Salmonella* Typhimurium cultivated in vitro and the antibody response of animals infected with live bacteria.

Based on this, we introduced a method for discovering *Salmonella* proteins able to induce condition-specific antibody production, which allows us to serologically distinguish animals that were vaccinated from those infected with *Salmonella* Typhimurium.

## Methods

### Bacterial strain

*Salmonella enterica* serovar Typhimurium phage type DT104 strain (strain number 1A5, from bacterial collection at Veterinary Research Institute, originally isolated from healthy sow), hereinafter referred to as *Salmonella* Typhimurium, was used in this experiment. Bacteria were cultivated overnight at 37 °C in Miller's LB Broth Base (Invitrogen, USA) or brain heart infusion (BHI) broth (Oxoid, UK) for certain analyses as described below.

### Vaccination and infection of pigs

Three groups of animals consisting of twelve white mixed-breed piglets (bought from a commercial stud) weaned 21 days after birth were used in the experiment. Pigs in the first group remained serologically negative for anti-*Salmonella* antibodies (determined by Pig Screen ELISA, Qiagen, Germany). Another twelve animals were orally infected one week after housing with 1 × 10^8^ CFU of *Salmonella* Typhimurium grown in BHI medium and blood was collected 28 days after the infection. The last group of animals was vaccinated intramuscularly into the neck with 1 ml of a vaccine prepared from 1 × 10^9^ CFU of *Salmonella* Typhimurium grown in BHI medium, inactivated with formaldehyde and adjuvanted with Montanide ISA50V2 (Seppic, France). The first dose was administered one week after housing and the second dose two weeks later. Blood was collected 14 days after the second dose of a vaccine.

### Antibody fraction preparation

Serum samples from three random animals from each group were pooled together and IgG fractions were isolated using Protein G columns (HiTrap Protein G HP, GE Healthcare, UK) according to the manufacturer’s protocol. Affinity chromatography was performed on an FPLC instrument (Pharmacia, Sweden).

### Antigen preparation

The same procedure of preparing bacterial protein lysate was used for the comparison of *Salmonella* Typhimurium protein expression when cultivated in LB or BHI medium using MS and for immunoaffinity chromatography.

*Salmonella* Typhimurium was grown overnight at 37 °C in LB and BHI medium, as noted above. The culture was then centrifuged (3,500 × g, 10 min), and a cell pellet was washed 3 times in PBS (Dulbecco’s, Lonza, Switzerland). The cell pellet was resuspended in PBS and sonicated (Sonopuls HD 3100, Bandelin, Germany) with zirconia/silica beads (BioSpec Products, USA). The sonicate was centrifuged at 20,000 × g and the supernatant with proteins was taken. The pellet was then resuspended in 8 M urea (Serva, Germany), 0.1 % SDS (Carl Roth, Germany), 2 % Triton X-100 (Serva, Germany) and 25 mM triethylammonium bicarbonate (Sigma-Aldrich, USA) and centrifuged at 20,000 × g again. The supernatant was mixed with the supernatant from the previous step and together used as an antigen for subsequent analysis.

### Immunoaffinity chromatography

IgGs from all three sample groups were covalently bound to CNBr-activated Sepharose (GE Healthcare, UK) according to the manufacturer’s protocol. Sepharose with bound immunoglobulins was loaded into 3 plastic cartridges (BioRad, USA) creating liquid chromatography columns. The same antigen (protein lysate from *Salmonella* Typhimurium grown in LB) was used for all three IgG columns - column with antibodies from negative control animals, from vaccinated animals and from infected animals. 20 mM sodium phosphate buffer, pH 7.0 (Serva, Germany) served as a binding buffer. 0.1 M glycine-HCl (Serva, Germany), pH 2.7 was used for elution of bound proteins and 1 M Tris–HCl (Carl Roth, Germany), pH 9.0, was added to collection tubes for pH adjustment. Protein eluates were then used for analysis by mass spectrometry.

### LC-MS/MS analysis

100 μg of proteins (measured with Pierce™ BCA Protein Assay Kit, Thermo Scientific, USA) was used for each sample preparation with the FASP (filter-aided sample preparation) method [10]. Each sample was washed five times with 8 M urea (Serva, Germany) in Vivacon 500 centrifugal tubes (Sartorius Stedim, Germany) with 10,000 MWCO membrane filter. Dithiothreitol (10 mM, Sigma-Aldrich, USA) and iodoacetamide (50 mM, Serva, Germany) in triethylammonium bicarbonate buffer (25 mM, Sigma-Aldrich, USA) were used for reduction and alkylation, respectively. Proteins were then digested with trypsin (Promega, USA) in 1:50 ratio, for one hour, at 37 °C and then overnight at 25 °C. After centrifugation, the eluate with digested peptides was evaporated (DNA120 SpeedVac, Thermo Savant, USA), peptide pellet was resuspended in 0.1 % aqueous formic acid (Sigma-Aldrich, USA) which serves as a mobile phase for liquid chromatography (UltiMate 3000 RSLCnano, Dionex -Thermo Scientific, USA). For separation and elution of peptides, 2-h gradient with increasing (0 min - 4 %, 4 min - 4 %, 98 min - 45 %, 98.5 min - 90 %, 112 min - 90 %, 112.5 min - 4 %, 120 min - 4 %) concentrations of acetonitrile (0.1 % formic acid in acetonitrile, Sigma-Aldrich, USA) at a flow rate of 300 nl/min was used. Peptides were separated on a 25 cm column (Acclaim PepMap RSLC C18, 2 μm, 100 Å, 75 μm I.D., Thermo Scientific, USA). uHPLC was connected to EASY-Spray ion source and Orbitrap Velos Pro mass spectrometer (Thermo Scientific, USA). A survey scan over the m/z range 390–1700 was used to identify protonated peptides with charge states of at least 2, which were automatically selected for data-dependent MS/MS analysis and fragmented by collision with helium gas. Ten fragment mass spectra after each full scan were recorded. Measured spectra were then searched using Proteome Discoverer (version 1.4, Thermo Scientific, USA) software with Mascot (Matrix Science, USA) or Sequest HT (Thermo Scientific, USA) as a searching algorithm. Oxidation of methionine as a dynamic, and carbamidomethylation of cysteine as a static modification was used. Precursor and fragment mass tolerances were set up as 10 ppm and 0.5 Da, respectively. Swiss-Prot database for *Salmonella* strain was used (based on 2014/06 release) in Mascot and Uniprot database for *Salmonella* strain (from 2013/04) was used in Sequest HT. Only peptides with a false discovery rate less than 0.05 were considered as well identified. Proteins that were uniquely found in each sample - proteins bound to the IgG fraction from sera from either vaccinated or infected animals, but not from control pigs were then pinpointed as candidate antigens for serological discrimination.

### Recombinant protein preparation and its purification

Appropriate primers (Tab. 1) for each selected protein were designed using NCBI/Primer-BLAST. PCR reaction with *Salmonella* DNA (supernatant from washed cell culture heated in distilled water for 10 min at 95 °C) as a template and Taq polymerase (Plain PP Master Mix, Top Bio, Czech Republic) was carried out. PCR products were purified (MinElute PCR Purification Kit, Qiagen, Germany), then cloned into the pTrcHis-TOPO vector (Invitrogen, USA) and recombinant proteins were expressed in TOP10 One Shot chemically competent *E. coli* cells (Invitrogen, USA). IPTG was used as an inducer of the expression. Recombinant proteins with N-terminal His-Tag were isolated on HiTrap Chelating HP columns (GE Healthcare, UK) with attached nickel ions. Briefly, an overnight *E. coli* culture in LB broth (Invitrogen, USA) was centrifuged and washed in PBS (Dulbecco’s, Lonza, Switzerland) and cells were disrupted by sonication (Sonopuls HD 3100, Bandelin, Germany). After centrifugation, supernatant with proteins was transferred to a binding buffer (0.02 M sodium phosphate, 0.5 M NaCl, 5 mM imidazole, pH 7.4) using desalting columns (PD-10, Sephadex G-25 M, GE Healthcare, UK). Metal-chelate affinity chromatography was performed on an FPLC system (Pharmacia, Sweden). After washing with a buffer containing an increased concentration of imidazole (40 mM), His-Tagged proteins were finally eluted with the elution buffer (0.02 M sodium phosphate, 0.5 M NaCl and 250 mM imidazole, pH 7.4).

**Table 1:** Primer pairs of DNA sequences for used proteins (or polypeptide fragments) and their expected molecular weight including HisTag

Protein	Forward primer 5′-3′	Reverse primer 5′-3′	Expected protein molecular weight
BamB	ATGCAATTGCGTAAATTACTTCTGCC	TTAACGCGTAATCGCGTAGACC	45.8 kDa
FliC-frag	ACGCTGAATGTGCAACAAAAATATAAGGTC	ACCTTCGGCTTTACTTGCAGCG	25.8 kDa
MetQ	ATGGCGTTCAAATTCAAAACCTTTGCGG	TTACCAGCCTTTCACCGCGCC	33.3 kDa
PrgI	ATGGCAACACCTTGGTCAGGCTATC	TTAACGGAAGTTCTGAATAATGGCAGCATC	12.7 kDa
SipC	ATGTTAATTAGTAATGTGGGAATAAATCCCGCC	TTAAGCGCGAATATTGCCTGCGAT	46.8 kDa
SipD	ATGCTTAATATTCAAAATTATTCCGCT	TTATCCTTGCAGGAAGCTTTT	41.0 kDa
SseB	ATGTCTTCAGGAAACATCTTATGGGGAA	TCATGAGTACGTTTTCTGCGCTATC	25.4 kDa
SurA	ATGAAGAACTGGAAAACGCTGCTTC	TTAGTTACTCAAAATCTTAACGTAAGCGCTG	51.1 kDa
SipB	ATGGTAAATGACGCAAGTAGCATTAGCCG	TTATGCGCGACTCTGGCGCAGAATAAAAC	66.3 kDa
OppA	ATGTCTAACATCACGAAAAAAAGTTTG	TTAATGTTTGATAATATATAAGTTTTTCACATAAAT	65.1 kDa

If further purification was needed, we performed a preparative electrophoresis in a polyacrylamide gel (Model 491 Prep Cell, BioRad, USA) according to the manufacturer’s protocol. For protein expression and purity verification, SDS-PAGE and western blot with a mouse anti-HisTag antibody (Pierce, Thermo Scientific, USA) was used.

### Western blot

Purified recombinant proteins were resolved on a 10 % SDS-PAGE gel and transferred to PVDF membranes (Amersham, GE Healthcare, UK), which were subsequently blocked in 1 % casein hydrolysate (Imuna, Czech Republic) overnight at 4 °C. The membranes were incubated with 100× diluted porcine sera for one hour at room temperature (RT), washed in PBS with 0.05 % Tween-20 (PBS/T, Serva, Germany) and then incubated (one hour, RT) with goat anti-pig IgG conjugated with HRP (Bethyl Laboratories, USA). After a washing step, the protein bands were visualized with diaminobenzidine (Sigma-Aldrich, USA). Western blots were performed with group-specific pooled sera samples.

### ELISA assay

Purified recombinant proteins in 0.05 M sodium carbonate-bicarbonate buffer (pH 9.6) were coated (4 °C overnight) on microtiter plates (MaxiSorp, Nunc, Denmark) that were blocked afterwards with 1 % casein hydrolysate in PBS/T (30 min). The plates were subsequently incubated (one hour, RT) with 100× diluted porcine sera, washed with PBS/T and then incubated (one hour, RT) with goat anti-pig IgG conjugated with HRP (Bethyl Laboratories, USA). After a washing step, the colour was developed by adding the chromogenic substrate TMB Complete (Test-line, Czech Republic) and the reaction was stopped with 2 M sulfuric acid (Penta, Czech Republic). The absorbance was measured at 450 nm on Synergy H1 (Biotek, USA). IgG antibody response to LPS was determined by commercially available Pig Screen ELISA (Qiagen, Germany). The individual serum samples were tested in duplicate by ELISA. Positive thresholds in time course graphs were counted as an average plus three standard deviations of all control samples and time zero samples of respective groups [11]. Positive thresholds for LPS commercial kit were provided by a manufacturer.

### Statistical evaluation

Densities of western blot bands were calculated using ImageJ software (ver. 1.48). Differences between groups in ELISA assays were evaluated by ANOVA with Dunn’s post test using GraphPad Prism (ver. 5). All graphs were also made using GraphPad Prism software (GraphPad Software, USA).

## Results

### Protein expression profiles in different media

At first, we examined protein expression profiles of *Salmonella* growing in different cultivation media. LB broth was used as a standard growth medium for cultivation of bacteria. For comparison, brain heart infusion (BHI), a highly nutritious medium was used as a vaccine preparation medium when the vaccine was proved to be protective [9]. From the whole-proteome mass spectrometry analysis, we observed the differences in LB and BHI cultures in relative quantities of several proteins that belong to the SPI-1 and SPI-2 loci which are important for the virulence of *Salmonella.* Relative quantification was attained using spectral counting and, in Tab. 2, it is shown as PSMs (peptide spectrum matches) for all peptides which identify the protein. Since PSMs depend on the length of a protein, the comparison of PSM value for the same protein between each medium is more informative rather than the absolute number. Note that the shown proteins are only representatives of the SPI-1 and SPI-2 loci and not the complete list.

**Table 2:** Several selected proteins belonging to SPI-1 and/or SPI-2 locus and their relative amounts produced by *S*. Typhimurium grown in LB and BHI medium, respectively

Locus	Protein	Accession number	PSMs	
			LB	BHI
SPI-1	SipA	P0CL52	37	2
SPI-1	SipB	Q56019	31	4
SPI-1	SipC	P0CL47	50	3
SPI-1	SipD	Q56026	10	0
SPI-1	PrgI	P41784	5	2
SPI-1	SptP	P74873	20	0
SPI-1	SopA	Q8ZNR3	16	5
SPI-1	SopB	O30916	38	0
SPI-1	SopE2	Q7CQD4	5	0
SPI-1 and-2	SteA	Q8ZPD7	0	0
SPI-1 and-2	SopD	P40722	5	0
SPI-2	SopD2	Q8ZQC8	3	0
SPI-2	SseB	Q7BVH7	0	3
SPI-2	PipB	Q8ZQ59	0	1
SPI-2	PipB2	Q8ZMM8	4	0
flagellar proteins	FliC	P06179	132	77
	FljB	P52616	107	61
reference proteins	PepN	Q8ZQ76	48	39
	GapA	P0A1P0	185	279

All selected SPI-1 proteins were expressed in much higher amounts in LB medium than in BHI medium while proteins belonging to the SPI-2 locus were not highly expressed in either of the media. Flagellar proteins that also play an important role in the first stages of an invasion were expressed in both media in high amounts. Proteins PepN (Aminopeptidase N) and GapA (Glyceraldehyde-3-phosphate dehydrogenase A) are mentioned only as reference proteins.

### Mass spectrometry analysis of immunoaffinity chromatography samples

*Salmonella* protein lysates preparations and consecutive immunoaffinity chromatography analyses resulted in three sets of samples for MS identification (*Salmonella* proteins bound to antibodies from control, infected or vaccinated animals). While most of the proteins were identified in all three samples in similar proportion, we found several proteins that were identified in relatively higher amounts in “post-infection” or “post-vaccination” fraction (Tab. 3). Relative amounts are shown as peptide spectrum matches (PSMs). Proteins SipB and SipC from SPI-1 were found in eluates from the column with post-infection sera while several proteins (OppA, BamB and SurA) were predominantly bound to antibodies in post-vaccination sera. We also included the identification of proteins that were chosen for their involvement in the infection process (SipD, SseB, PrgI and FliC) rather than from immunoaffinity chromatography/MS analysis only.

**Table 3:** Identification and relative quantification of chosen proteins in terms of Peptide Spectrum Matches for all three runs

Protein name	Accession number	PSMs (peptide spectrum matches)		
		Control	Post-infection	Post-vaccination
Cell invasion protein SipB	Q56019	3, 0, 22	29, 20, 27	13, 7, 25
Cell invasion protein SipC	P0CL47	5, 1, 22	30, 33, 31	7, 18, 26
D-methionine-binding lipoprotein MetQ	Q8ZRN1	3, 1, 0	12, 5, 11	0, 0, 9
Periplasmic oligopeptide-binding protein OppA	P06202	7, 0, 0	31, 0, 0	25, 16, 14
Outer membrane protein assembly factor BamB	H9L451	0, 2, 0	0, 0, 7	7, 10, 0
Chaperone SurA	Q7CR87	1, 5, 1	2, 0, 21	21, 15, 15
Cell invasion protein SipD	Q56026	7, 3, 0	4, 15, 0	4, 14, 1
Secreted effector protein SseB	Q7BVH7	0, 0, 0	2, 0, 0	1, 0, 1
Protein PrgI	P41784	0, 2, 0	1, 2, 0	2, 0, 1
Flagellin FliC	P06179	3, 22, 17	23, 29, 26	19, 17, 63

### Recombinant protein preparation

**Fig. 1:**
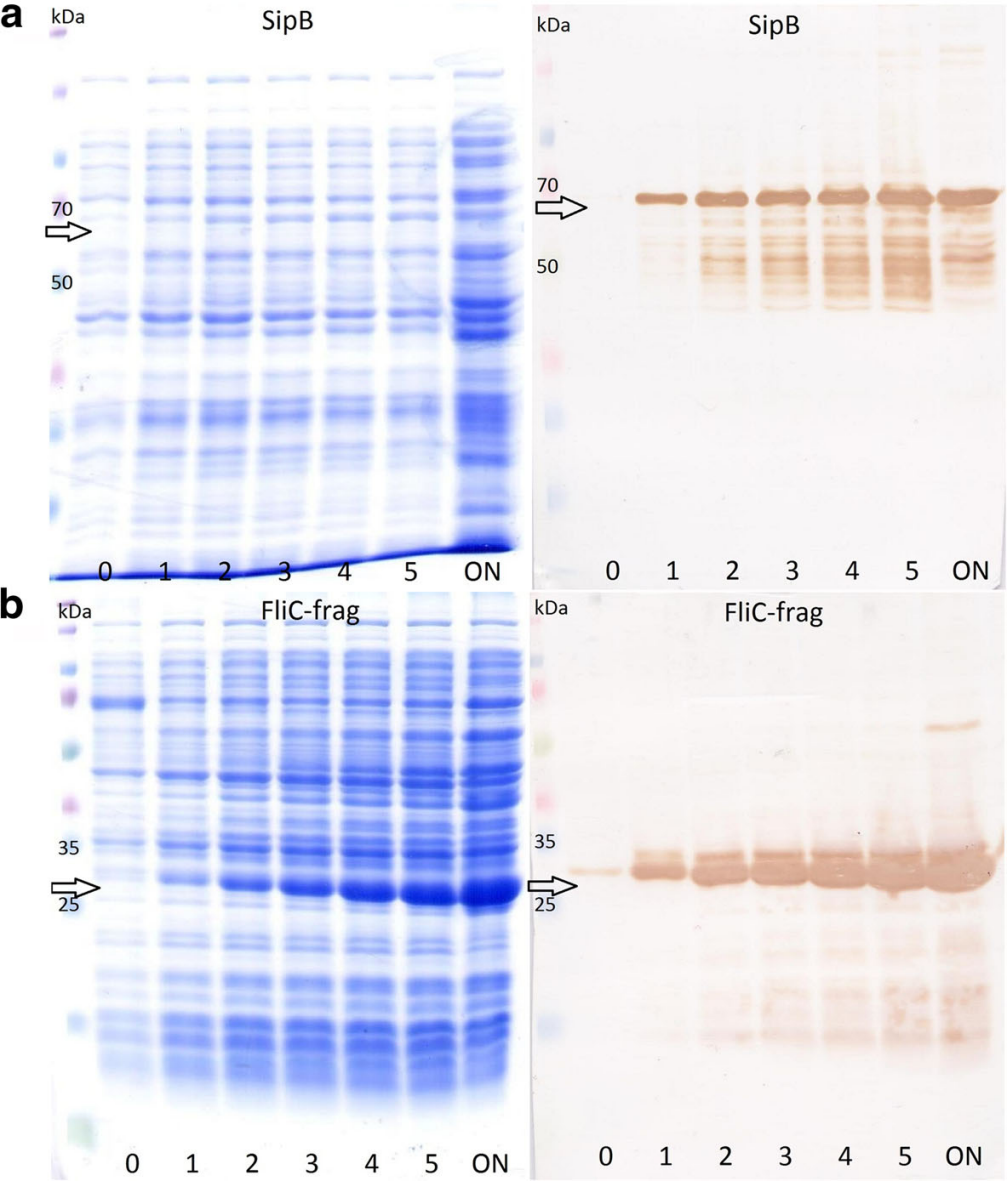
Recombinant proteins expressions. Coomassie-stained gels and western blots of SipB (**a**) and FliC-frag (**b**) proteins. Time scale expression is shown in hours post induction (0–5 h, ON - overnight). Arrows indicate the expected molecular weight of a product. Expression of other proteins can be found in Additional file 1: Figure S1

All proteins listed in Table 3 were prepared as recombinant proteins in an *E. coli* expression system. The sequences of all proteins containing signal peptides at their N-termini were designed without this peptide. Otherwise, they possessed an identical sequence as naturally occurring in *S*. Typhimurium except FliC protein. Due to an inability of full-length protein expression, flagellar protein FliC was expressed only as a 22 kDa fragment. The expression of proteins was endorsed by SDS-PAGE and confirmed by western blot with an anti-HisTag antibody (Fig. 1 and Additional file 1: Figure S1). Four out of ten selected proteins (MetQ, SurA, PrgI, SipC) were not expressed in sufficient amount or purity and were not included in subsequent serological testing.

### Serological testing

#### Western blot

**Fig. 2:**
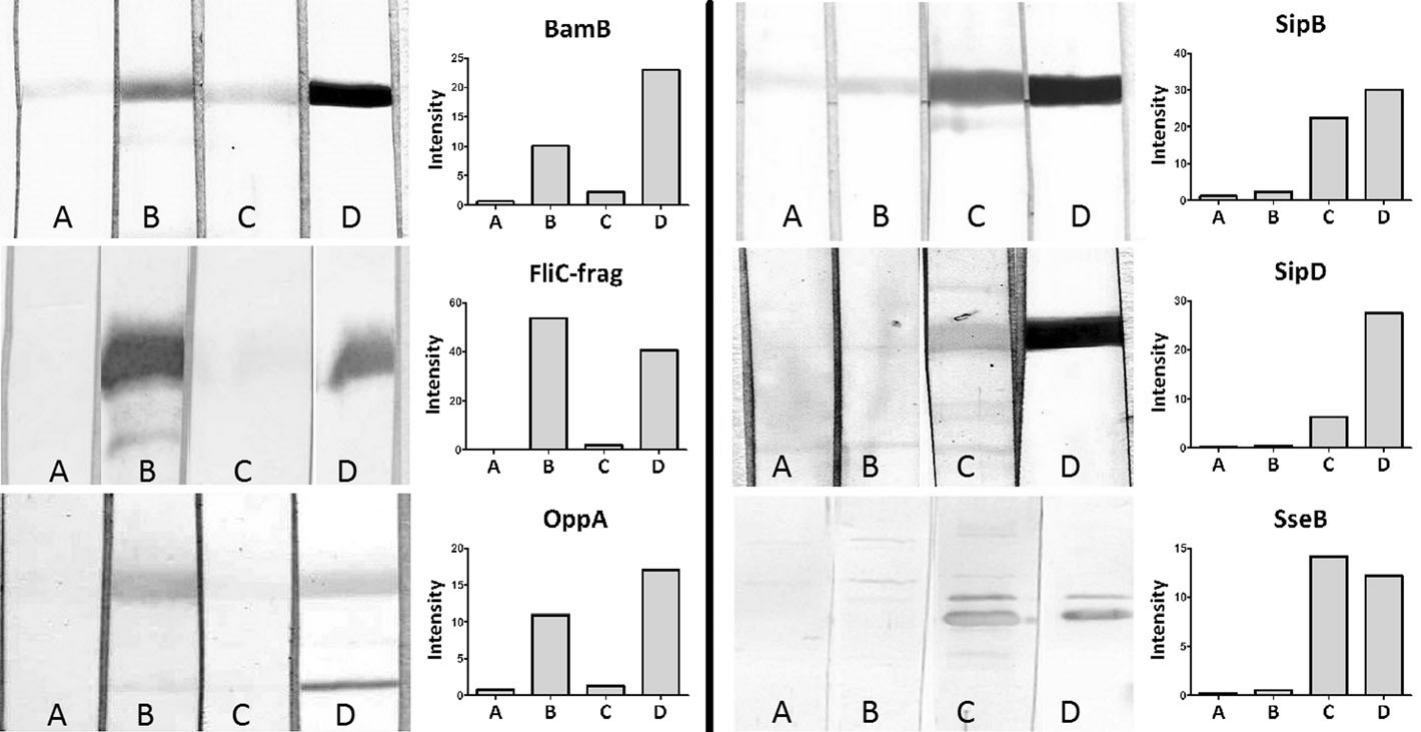
Western blots of recombinant proteins with sera. Western blots with respective density graphs of purified recombinant proteins with **A** – control sera, **B** – post-vaccination sera, **C** – post-infection sera, and **D** – with anti-HisTag Ab

Purified recombinant proteins were then used as antigens for serological analysis. After western blot, PVDF membranes were incubated with pooled serum samples from three animals of each group (control, infected and vaccinated animals). As shown in Fig. 2, proteins SipB, SipD and SseB presumed to be “post-infection” antigens were detected by sera of infected animals with a higher intensity than by sera of control or vaccinated animals. On the other hand, proteins BamB, FliC-frag and OppA, supposed to be “post-vaccination” antigens, were detected by sera of vaccinated pigs with a higher intensity than by sera of control or infected animals. Anti-HisTag antibody was used as a positive control for recombinant protein presence (in Fig. 2D).

#### ELISA assay

Based on western blot results, proteins SipB and FliC-frag were selected for testing specific antibody response of individual animals by ELISA method. Serum samples from 12 animals in each group were used for measuring antibody levels against recombinant SipB and FliC-frag proteins.

Antibody levels against SipB protein in sera of control animals had the values of absorbance between 0.062 and 0.126; sera of vaccinated animals had the values of absorbance between 0.075 and 0.147. The difference in absorbance between these two groups was not statistically significant. Sera of infected pigs had the values of absorbance between 0.248 and 1.056. These values were significantly different (P <0.01) from the two previous groups of animals.

**Fig. 3:**
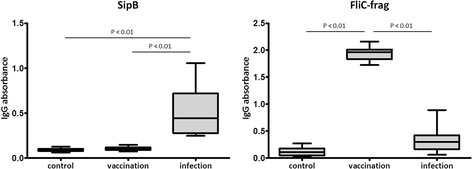
ELISA assay of recombinant proteins. Graphs of IgG absorbance from ELISA assays of two recombinant proteins incubated with individual sera of control animals (*N* = 12), STM infected animals (*N* = 12) and animals after vaccination with STM-based vaccine (*N* = 12)

Antibody levels against FliC-frag proteins in sera of control animals had the values of absorbance between 0.018 and 0.271; sera of infected animals had the values of absorbance between 0.060 and 0.891. The difference in absorbance between these two groups was not statistically significant. Sera of vaccinated pigs had the values of absorbance between 1.727 and 2.159. These values were significantly different (P <0.01) from two previous groups of animals (Fig. 3). Hence, proteins SipB and FliC-frag may serve as marker proteins applicable for serological discrimination between vaccinated pigs, pigs after natural infection and pigs without any contact with *S*. Typhimurium.

**Fig. 4:**
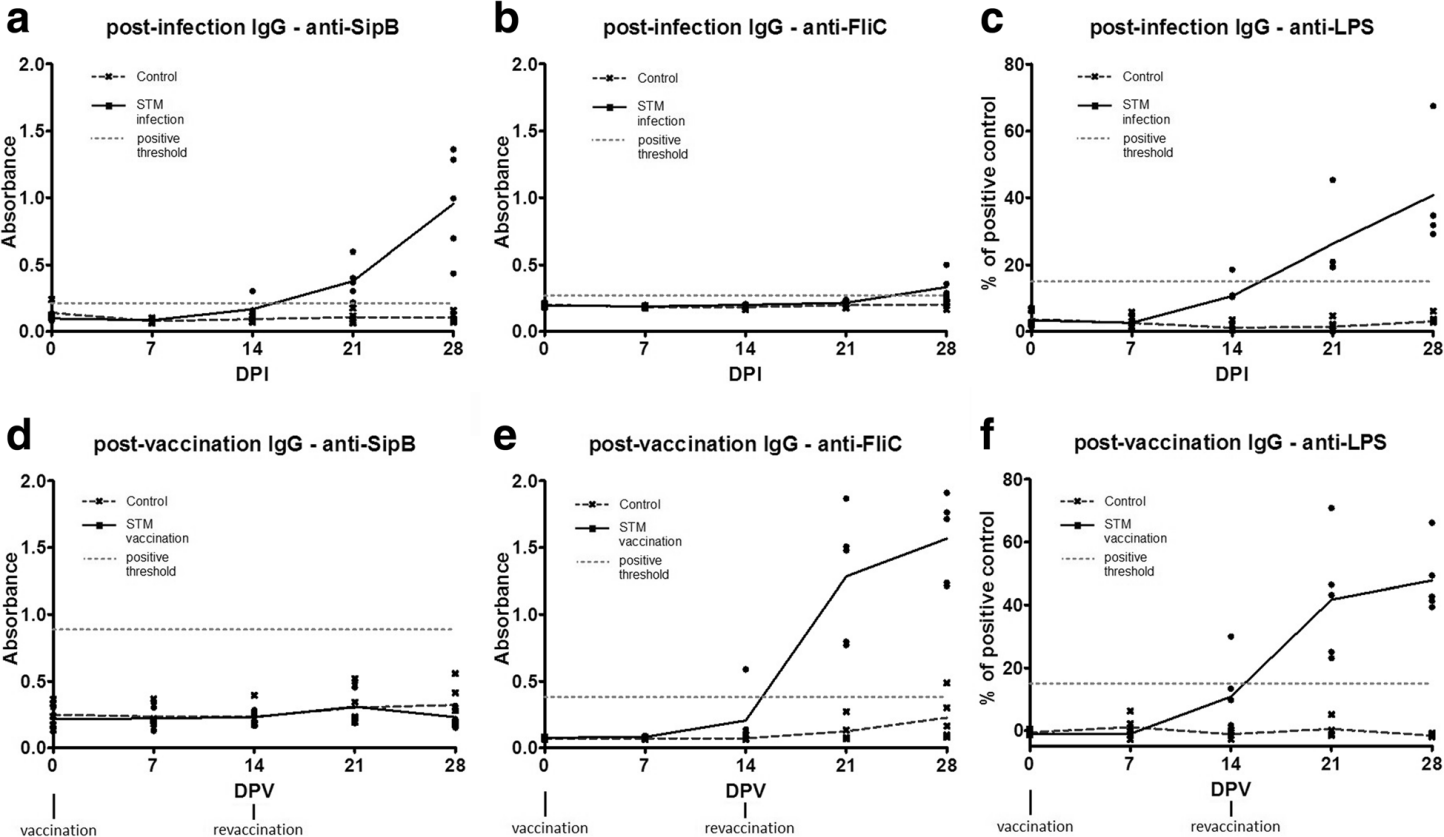
Time course of antibody response. The time course of IgG antibody response to SipB and FliC-frag proteins after the infection (**a, b**) or vaccination (**d, e**). For comparison, graphs **c** and **f** show antibody response to O-antigens of *Salmonella* LPS. The connecting lines determine the average of five animals randomly selected from each group

When compared to a non-infected group, from graphs showing the time course of antibody response (Fig. 4) we can see the increase of anti-SipB IgG level from the third week of infection but not significant differences for anti-FliC antibodies throughout the infection experiment (Fig. 4a, b). In contrary, the vaccination does not induce anti-SipB antibody production but anti-FliC IgGs are detectable after 14 days that is the time of revaccination (Fig. 4d, e). For comparison, we measured the level of post-infection and post-vaccination antibodies against O-antigens - the outer segments of surface lipopolysaccharides using commercially available ELISA kit (Fig. 4 c, f). As expected, we found a similar pattern of immunoglobulin G production in both, infected and vaccinated groups. Additionally, a concentration of IgM antibodies against SipB protein did not exceed positive threshold after the infection. Anti-LPS IgMs were detectable on day 7 and 14 in two and four pigs respectively (see Additional file 1: Figure S2).

## Discussion

Although the vaccination of animals against bacterial pathogens helps to moderate the severity of infection and is widely used in veterinary medicine, there are several issues that have to be solved during the development process and before introducing such vaccines to the market. Besides the induction of humoral and cellular immunity - the protectivity itself, in many cases, we must ensure a proper distinction between animals after the natural infection and vaccinated animals – i.e. DIVA vaccines [12]. We believe that using a vaccine with differentiability of infected and vaccinated animals enable establishment of *Salmonella* control programs in pig herds. This goal is currently not possible using the only licensed live attenuated vaccine [7]. In a case of *Salmonella* DIVA vaccines, several groups followed the path of a negative marker vaccine [7, 8], which uses a vaccine strain with a deleted gene for an antigenic protein. Host antibodies against this protein are thus found only in naturally infected animals and are absent in vaccinated animals. However, using live attenuated genetically modified *Salmonella* vaccine strains in pig herds is not straightforward. We proved inactivated *Salmonella* vaccine efficiently protects suckling piglets against infection [9] and further developed a DIVA method whereby there is no need for using a live genetically modified organism as an antigen for a vaccine. To develop DIVA assay, we decided to use a novel approach based on examination of the natural antibody response against *Salmonella* Typhimurium after the infection and after the vaccination with a *Salmonella* Typhimurium-based inactivated vaccine. We took advantage of that *Salmonella* Typhimurium undergoes several different stages during the infection according to its localization and to the host immune response. Following these conditions, bacteria change the expression of virulence and protecting proteins [13, 14] that are subsequently exposed to the immune system.

Besides proven protectivity of the used vaccine in suckling piglets [9], we found that antibodies produced after the infection, are indeed different from those after the vaccination. From the immunoaffinity chromatography and mass spectrometry, we selected the following candidate proteins – SipB, SipC, MetQ, OppA, Bam and SurA. Then, we added some proteins that were not determined by the aforementioned method, but are known to be involved in the infection process as they belong to the SPI-1 or SPI-2 locus – SipD, SseB and PrgI and a highly immunogenic flagellar protein FliC.

Proteins SipB, SipC and SipD are representatives of the SPI-1 system which is responsible for invading the host cells. It is well established that these proteins form a tip of a needle structure of the type III secretion system complex that interacts with the eukaryotic cells [13, 15]. Protein SseB encoded by the SPI-2 locus acts as a translocator that mediates translocation of effector proteins into the host cells and promotes bacterial survival [16]. These proteins are therefore crucial for virulence and are candidates for inducing the antibody response that was also confirmed in this work. On the other hand, when *Salmonella* Typhimurium was cultivated *in vitro* in BHI culture medium as a vaccine batch, these proteins were not expressed in amounts able to induce antibody production. SPI-1 proteins were nevertheless found in high amounts when *Salmonella* had grown in LB medium. This medium is therefore not applicable for a vaccine batch preparation because it could induce production of the same antibodies as after a natural infection. Nutrient-limiting conditions provided by LB broth partially resemble *in vivo* environment and trigger SPI-1 proteins expression, while rich BHI medium does not induce production of *Salmonella* virulence factors. Jaradat and Bhunia [17] suggested a similar explanation as gained from their results in different growth conditions of *Listeria monocytogenes,* stating that bacteria produce more virulence factors in a nutritionally poor milieu in order to adapt to the new environment. On the other hand, bacterial virulence factors seem to be good candidates for antibody response induction and therefore for the vaccine protectivity in general. However, we showed that the efficacy of our vaccine prepared from a culture grown in a medium not inducing the expression of SPI proteins is high [9].

In spite of the fact that flagellin has powerful immunostimulatory properties and may play a role as a potent adjuvant in a variety of anti-bacterial vaccines [18–20], it was also shown that expression of flagellar proteins is selectively repressed when *Salmonella* is located intracellularly during the infection [21, 22]. The flagellum as an extracellular structure on the surface of a bacterium could be a good candidate as a post-vaccination antigen if an appropriate growth medium capable of promoting the expression of flagellar proteins is used. This was proved for FliC protein fragment by western-blot and ELISA but interestingly not by PSM spectra of immunoaffinity eluates. We speculate this was caused by overall high amount of anti-FliC antibodies in all samples and the capacity of immunoaffinity columns was probably saturated. Only western-blot and ELISA were able to show much higher anti-FliC antibody level in post-vaccination sera than in post-infection and control sera. PSM for FliC in control sample was probably caused by cross-reactivity of anti-flagellar antibodies induced by other bacteria in control animals. The other possible explanation is very high expression of FliC protein in cultures and possible unspecific binding in the chromatography column. Proteins BamB (Outer membrane protein assembly factor) and OppA (Periplasmic oligopeptide-binding protein) are located on the outer membrane and the periplasm, respectively, which makes them easy targets for the immune system. Although both proteins are involved in transmembrane transport (OppA as a permease [23] and BamB as a part of the outer membrane protein assembly complex involved in insertion of beta-barrel proteins [24]), their role in the infection is not known. Interestingly, both proteins are much less immunogenic during infection than after vaccination. This finding makes them together with FliC good candidates for serological differentiation between infected and vaccinated animals.

After successful isolation of recombinant proteins, we tested them using western blot and ELISA with post-infection, post-vaccination and control serum samples to prove their ability to distinguish between infected and vaccinated animals. Proteins SseB, SipB and SipD were confirmed to be highly immunogenic during the infection. This is in agreement with previous findings [25, 26]. The level of antibodies against these proteins, measured as a density of bands on western blots, was, at least, ten times higher in a group of infected animals than in a group of control or vaccinated animals. On the other hand, proteins BamB, OppA and FliC-frag were confirmed to be highly immunogenic during the vaccination of pigs. The level of antibodies against these proteins was, at least, ten times higher in a group of vaccinated animals than in a group of control or infected animals. As in the case of FliC, there is a partial discrepancy with immunoaffinity chromatography results in the case of SseB protein when it showed a similar level of binding to post-infection and post-vaccination antibodies. However, it can be explained by low expression of SseB in cultivation media and thus possible incomplete saturation of all specific antibodies present in the chromatography column.

Finally, proteins SipB and FliC-frag were selected for ELISA assays to examine the interaction with 36 porcine serum samples. All 12 post-infection sera exhibited higher absorbance with the SipB protein than 12 control and 12 post-vaccination sera. In contrast, all 12 post-vaccination sera exhibited higher absorbance with the FliC-frag protein than 12 control and 12 post-infection sera. All differences were statistically significant (P <0.01). The time-dependent onset of IgG antibody levels against SipB after the infection, and against FliC after the vaccination imitates the increase of IgG against surface lipopolysaccharide. These two proteins are thus considered as useful antigens for serological differentiation of infected and vaccinated animals.

In general, induction of different protein expression profiles, their potential exposure to the immune recognition machinery and subsequent specific antibody production can be conveniently applied for the differentiation between infected and vaccinated animals without any intervention into the genome of an organism used as a vaccine strain.

## Conclusions

Using antibodies from previously vaccinated and infected animals and using immunoaffinity chromatography and mass spectrometry tools, we identified *Salmonella* proteins that induce post-infection and post-vaccination specific antibody production. Gene cloning and recombinant protein expression techniques were used for antigen preparation that allows serological distinction between infected animals and animals vaccinated with a S*almonella*-based inactivated vaccine.

This approach can be utilized in many other challenges where DIVA vaccine and a subsequent serological assay are required, especially when genetic modification of a vaccine strain is not desirable.

## Additional files

Additional file 1: Figure S1. Recombinant proteins expressions. Coomassie-stained gels and western blots of recombinant proteins. Time scale expression is shown in hours post induction (0–5 h, ON - overnight). Arrows indicate the expected molecular weight of a product. **Figure S2.** IgM antibody response. The time course of IgM antibody response to SipB protein and to *Salmonella* LPS after the infection. The connecting lines determine the average of five animals randomly selected from each group. (PDF 434 kb)
Additional file 2: Identified *Salmonella* proteins from different media. Mass spectrometry identification of *Salmonella* proteins and a comparison of expression between two cultivation media (LB vs BHI). (XLSX 433 kb)
Additional file 3: Identified proteins from immunoaffinity chromatography. Mass spectrometry identification of *Salmonella* proteins bound to control, post-infection or post-vaccination serum. Immunoaffinity chromatography was made in triplicates. (XLSX 913 kb)

## Abbreviations

BHI: Brain-heart infusion
CFU: Colony-forming unit
DIVA: Differentiation of infected and vaccinated animals
FASP: Filter-aided sample preparation
FPLC: Fast protein liquid chromatography
HRP: Horseradish peroxidase
LB: Lysogeny broth
LPS: Lipopolysaccharide
MS: Mass spectrometry
PSM: Peptide spectrum match
PVDF: Polyvinylidene difluoride
SPI: *Salmonella* pathogenicity island
TMB: 3,3′,5,5′-tetramethylbenzidine
uHPLC: Ultra-high pressure liquid chromatography
