# Supplementary material for: A proteomic approach to the development of DIVA ELISA distinguishing pigs infected with Salmonella Typhimurium and pigs vaccinated with a Salmonella Typhimurium-based inactivated vaccine
Source: BMC Vet Res. 2016 Nov 11;12:252. doi: 10.1186/s12917-016-0879-1 (PMC5106837; doi:10.1186/s12917-016-0879-1)

Supplementary Fig. 1 Coomassie-stained gels and western blots of recombinant proteins. Time scale expression is shown in hours post induction (0 - 5 hours, ON - overnight). Arrows indicate the expected molecular weight of a product.

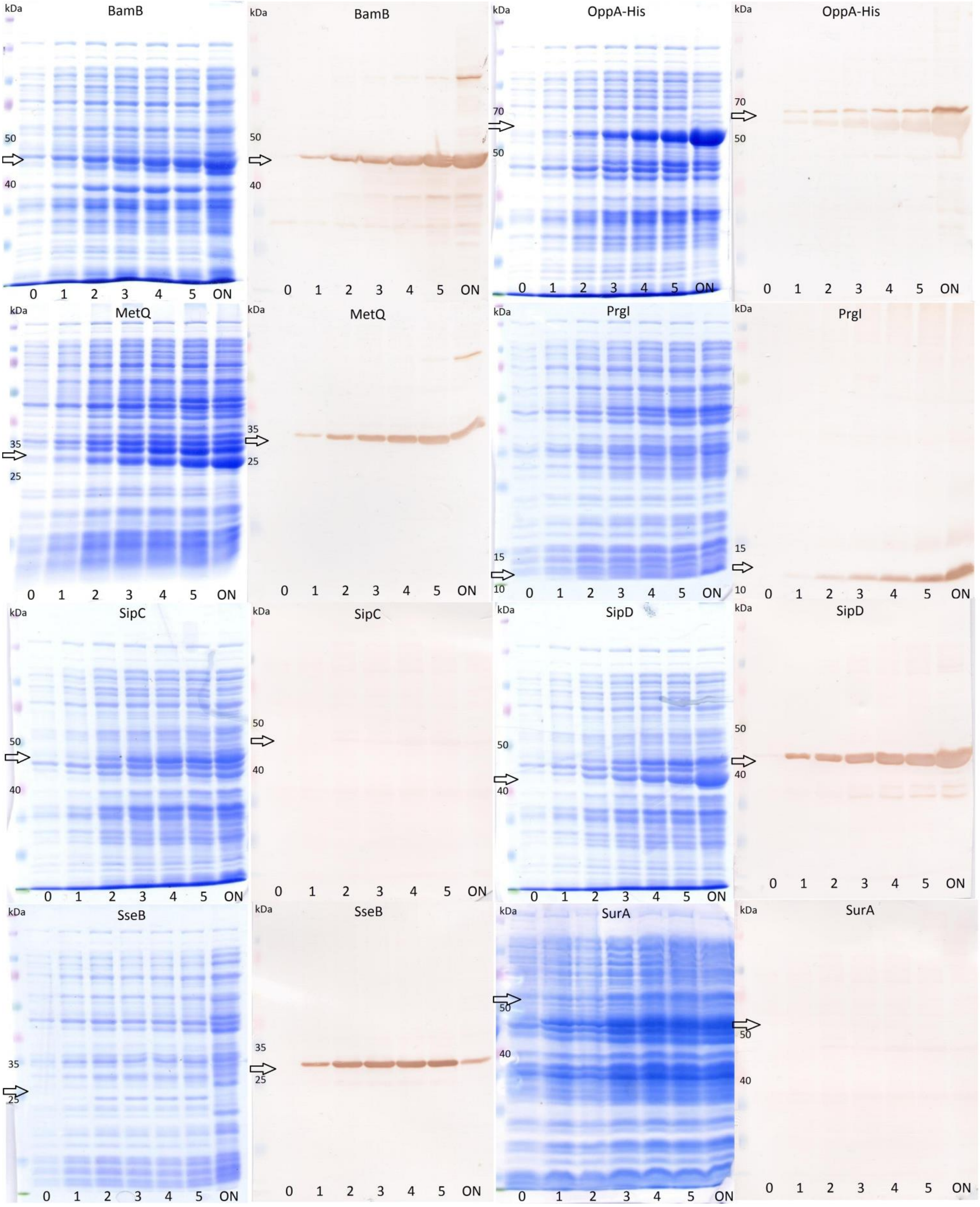

Supplementary Fig. 2 The time course of IgM antibody response to SipB protein and to Salmonella LPS after the infection. The connecting lines determine the average of five animals randomly selected from each group.

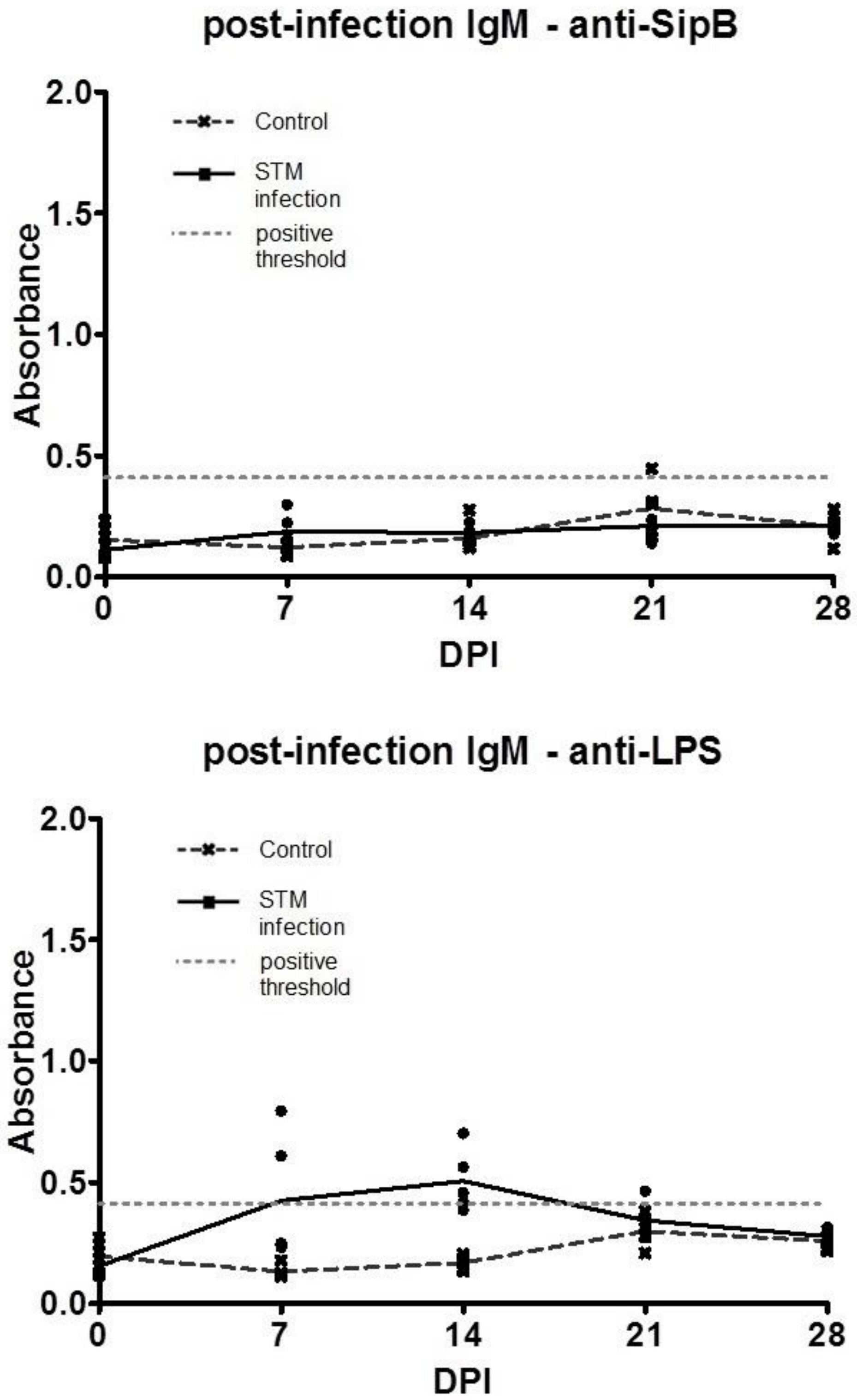

Supplement: Additional file 1: Figure S1. — Recombinant proteins expressions. Coomassie-stained gels and western blots of recombinant proteins. Time scale expression is shown in hours post induction (0–5 h, ON - overnight). Arrows indicate the expected molecular weight of a product. Figure S2. IgM antibody response. The time course of IgM antibody response to SipB protein and to Salmonella LPS after the infection. The connecting lines determine the average of five animals randomly selected from each group. (PDF 434 kb) [file 12917_2016_879_MOESM1_ESM.pdf]
